# Are orthopaedic surgeons prepared? An analysis of severe casualties from the 2021 flash flood and mudslide disaster in Germany

**DOI:** 10.1007/s00068-022-01967-2

**Published:** 2022-04-15

**Authors:** Martin Gathen, Kristian Welle, Max Jaenisch, Adnan Kasapovic, Charlotte Rommelspacher, Suncana Novosel, Jonas Roos, Koroush Kabir

**Affiliations:** Department of Orthopedics and Trauma Surgery, University Hospital of Bonn, Venusberg-Campus 1, 53127 Bonn, Germany

**Keywords:** Flood, Natural catastrophe, Infectious diseases, Trauma surgery, Natural disaster, Climate change

## Abstract

**Background:**

The purpose of this study was to describe and analyse the most severe casualties from the flash flood and mudslides occurring on 14 July 2021 in Germany, focusing on patients who were treated in the closest and largest level I trauma centre in the region the disaster occurred.

**Methods:**

A single-centre retrospective study design was employed, and all patients treated because of the flooding and mudslides who needed inpatient treatment were documented. Data on each patient’s demographic characteristics, type of injury, number of surgeries, duration of hospitalisation, operation time, revision rate, injury severity score (ISS), and complications were collected. The primary outcome measure was status at discharge.

**Results:**

Within the first week after the flood, a total of 63 patients were documented. Forty-one patients were treated on an outpatient basis in the emergency unit, and 22 patients were hospitalised. Of those hospitalised, 15 patients needed surgical treatment in the operation theatre. The most common injuries were fractures of the lower extremity (*n* = 7) and soft tissue wounds (*n* = 4). Overall, 20 surgeries were performed; the mean hospital stay was 7.2 ± 6.4 days, and the mean ISS was 5.7 ± 2.7.

**Conclusion:**

The July 2021 flood disaster was one of the largest in German history. The included patients showed complex injuries of various types. Because of the effects of climate change, orthopaedic surgeons might face higher numbers of casualties affected by natural disasters. Learning more about the management and profile of these injuries can become a future challenge for orthopaedic and trauma surgeons.

## Introduction

Natural disasters are major adverse events that result from natural processes and have devastating effects on living beings [1]. Events like hurricanes, floods, earthquakes, and wildfires can displace populations, destroy infrastructures, hinder economic growth, cause death and injury, and increase the risk of infectious disease outbreaks [2]. The characteristics and effects of disasters are becoming increasingly complex because of factors like climate change, urbanisation, economic interconnectivity and globalisation [2, 3]. Compared with a 1.2 °C cooler climate, the likelihood of such an event as the flash flood and mudslides in this study has increased by a factor between 1.2 and 9, suggesting that events like the one described will occur more frequently in the future [4].

To improve the care of seriously injured patients, the German Trauma Society founded the Trauma Network in 2008 [5]. The network consists of different levels. For a trauma network, at least one supra-regional, two regional and three local trauma centres must be present. The responsible trauma network consisted of 1 supra-regional, 4 regional and 4 local centres. As a level 1 centre, certified equipment features must be available and 24-h expertise must be guaranteed. All supra-regional trauma centres are committed to accepting patients who exceed the care capacities of smaller centres at any time.

On 14 July 2021, a large flood disaster unexpectedly hit parts of Germany. Because of persistent rainfall from the 12th to the 15th of July associated with a cut-off low-pressure system labelled ‘Bernd’, the states of Rhineland-Palatinate and North Rhine-Westphalia suffered severe flooding [4]. At least 220 people died across Europe, including 184 in Germany [6]. The *New York Times* referred to the floods in Europe as the latest signs of the global climate crisis [7]. A recent study supported this statement, showing that the observed rainfall amounts broke historically observed rainfall records by large margins and that such events are more likely to occur in a warmer climate compared with that in preindustrial times [4]. As a result of the rainfall, nearby hospitals were also affected: There was damage caused by the water, and some personnel simply could not reach the working space because of destroyed roads and infrastructure [8].

The goal of this study was to assess the types of injuries and complications of patients after a natural disaster in Germany and to share our experience in management and treatment of these patients. Literature about surgical care because of natural disasters in modern west Europe is rare. However, because of climate change and the resulting increase in events, the topic is likely to become more important for trauma and orthopaedic surgeons.

## Patients and methods

A single-centre retrospective study was conducted that included all victims of the flood disaster who were admitted to our hospital and sustained at least one musculoskeletal injury. Patients who were treated in the first week after the flood were included (14–21 July 2021). During this time, almost all patients directly affected by the disaster came to the hospital. Patients who had no injury and were treated exclusively for other symptoms, such as hypothermia or dehydration, were excluded. To analyse the data, the electronic patient file was used with the software "Orbis" (AGFA HealthCare, Mortsel, Belgium). Patients with minor injuries who could be treated on an outpatient basis were counted but not included in the analysis. The study was approved by the local institutional review board (University of Bonn Ethics Committee, No. 226/13).

After arriving at the hospital, all patients were classified into five levels of urgency using the German version of the Manchester Triage System (MTS). The system differentiates between immediate, very urgent, urgent, standard and non-urgent levels based on the priority [9]. Such parameters as oxygen saturation, blood pressure, heart rate, and body temperature were determined for each treated patient. For all patients with a status of immediate or very urgent, a FAST scan (Focused assessment with sonography for trauma) was performed. For each patient who received inpatient treatment, the following data were collected: age, sex, type of injury, whether treatment was surgical or non-surgical, duration of hospitalisation, and complications. To assess the severity of the injuries, the Injury Severity Score (ISS) was calculated. The ISS describes a clinical classification of injury patterns in which six body regions are examined (head and neck, face, chest, abdomen, extremities, and external injuries) and a score of 0–75 points is collected [10]. Severity is graded from 1 = minor to 6 = maximum using the Abbreviated Injury Scale (AIS). The ISS is then calculated using the following formula: ISS = (AISa)^2^ + (AISb)^2^ + (AISc)^2^. If a body region is classified with severity level 6, the ISS is always 75.

For each patient who received surgical treatment, additional data about the number of surgeries, operation time, blood loss, and microbiological specimens were collected. All fractures were classified according to the AO classification system [11].

The emergency centre normally consists of three shock rooms, six monitoring stations, and seven exam rooms. For patient care, parts of the outpatient clinic have been closed in the meantime, enlarging the emergency room and three additional rooms. Routine surgical care was paused on July 14th in the interim to create capacity for the injured. The mass casualty incident (MCI) protocol was not triggered because of the time-staggered arrival of the patients in the emergency room that could manage the number of patients well. Unlike an MCI with a singular event, such as a traffic accident, the situation was very dynamic. As the patients did not all appear at the same time, treatment and triage could proceed in an orderly manner, even if with a delay.

Given the psychological trauma of many patients, chaplains and the clinical crisis intervention team were called in to assist.

### Patient and public involvement

The research question was significantly influenced by the experiences and priorities of the patients. Numerous patients and relatives reported about the chaotic conditions after the flood with regard to communication, the organisation of logistical help and medical care [12, 13].

Since all patients included were treated as result of a natural disaster, it was not possible to involve them in the design of this study. No special measures are planned to forward the results to the participants.

## Results

The communication between hospitals, as is common for trauma network transfers, was almost impossible due to the lack of electricity and the collapsed mobile network.

Information exchange was therefore almost exclusively via the emergency services und was very limited. As a result, paramedics and emergency physicians triaged patients on scene to transport patients with more severe injury patterns to a Level 1 centre.

Due to the difficulty of rescuing patients on site, there were time delays in getting medical care to patients. Likewise, the arrival of many patients at the same time, led to a delay in the care of the lighter injured patients. Some of these had to wait several hours for their treatment in order to ensure the treatment of severely injured patients.

By reducing selective procedures and examinations, medical care could be compensated in the unaffected hospitals. Medical personnel stood by to be available to return to the hospitals in the event of an acute aggravation of the situation.

Several regional hospitals were partially or completely destroyed and patient care had to be suspended. Inpatients had to be transferred to the nearest hospitals and patient care was also limited in the following months. As a result, many patients were transferred to the Level 1 centre who were not direct members of the trauma network.

Sixty-three patients were treated in our clinic for orthopaedic and trauma surgery in the first week after the flood disaster. Forty-one patients had minor injuries that could be treated on an outpatient basis in the emergency unit. 30% of the patient belonged to the category very urgent and immediate. 10% belonged to the category urgent and standard. No patient belonged to the category not urgent.

Twenty-two patients were hospitalised (64% males, 36% females). Their mean age was 53.7 ± 18.9 years, and the mean hospital stay was 7.2 ± 6.4 days. Thirteen of these patients were injured during the flood, whereas the other nine patients suffered injuries during clean-up operations.

**Fig. 1:**
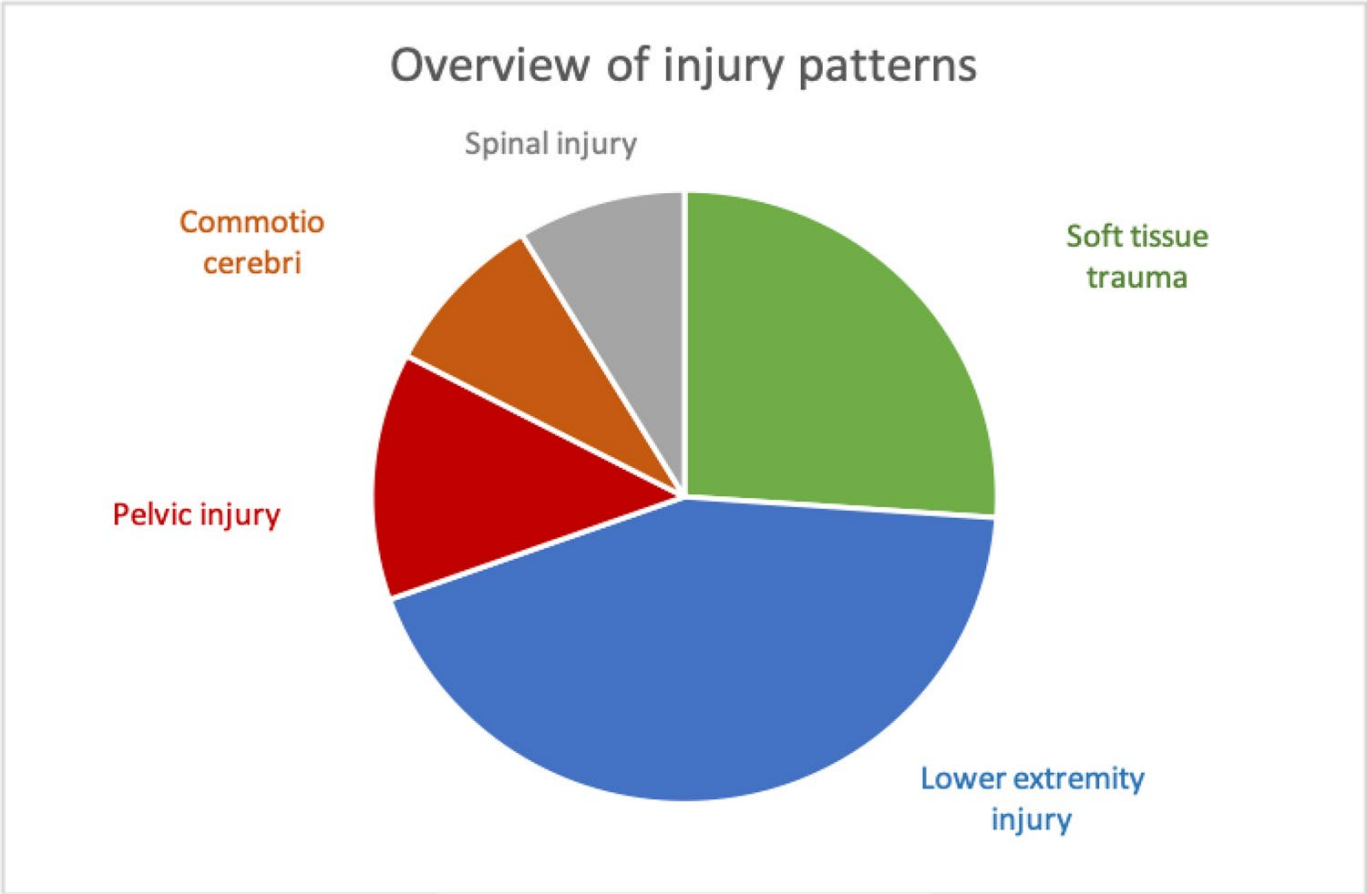
Injury patterns of all patients receiving inpatient treatment. In the case of multiple injuries, there are double entries

Seven patients received conservative treatment, whereas 15 patients needed surgical treatment. Overall, 20 surgeries were performed, with a mean operation time of 107 ± 94 min. The mean blood loss was 182 ± 236 ml, and one complication occurred. The patient developed pneumonia and required antibiotic treatment for several days. An overview of the quantitative data is shown in Table 1. The most common injuries leading to hospitalisation were fractures of the lower extremity (*n* = 7) and soft tissue wounds (*n* = 4). Figure 1 shows the distribution of the injuries. To objectively classify the injury severity, the ISS was measured, showing a mean ISS of 5.7 ± 2.7. This illustrates that no patient suffered a polytrauma.

**Table 1:** Descriptive summary of demographic characteristics, treatment, and the time of arrival to the hospital of all patients receiving inpatient treatment

Quantity	22
Male	*n* = 14 (64%)
Female	*n* = 8 (36%)
Age	53.7 ± 18.9
ISS	5.7 ± 2.7
Duration of stay (days)	7.2 ± 6.4
Number of Operations	0.86 ± 0.71
Operation time (min)	107 ± 94.2
Operative treatment	*n* = 15
Conservative treatment	*n* = 7
Arrival within 24 h after the disaster	*n* = 6
Arrival later than 24 h after the disaster	*n* = 16

In four cases, microbiological specimens were taken during surgery, and in three patients, a pathogen could be detected (Table 2). In all positive cases, an enterococcus species was found. All patients with soft tissue injuries and contact with water received Tazobactam as an empiric antibiotic. Tazobactam was chosen first because of its approval for soft tissue infections and its efficacy against enterococcus species.

**Table 2:** Summary of patient diagnostics and surgical treatment of patients receiving inpatient care

Patient no	Diagnosis	Number of operations	Surgery time (min)	Blood loss (ml)	Bacterial microbial layer	Complications	Antibiotics
Patient 1	Open wound plantar side right foot	1	27	50	–	–	Tazobactam
Patient 2	Periprosthetic femur fracture Vancouver B2	1	347	600	–	No	–
Patient 3	Periprosthetic femur fracture Rorabeck III	1	241	700	–	No	–
Patient 4	Soft tissue mixed infection	2	69	50	*Proteus mirabilis*, *Klebsiella pneumoniae*, *Klebsiella oxytoca*, *Escherichia coli*, *Aeromonas hydrophila*, *Enterococcus faecalis*, *Staphylococcus cohnii* subspecies urealyticus	No	Tazobactam
Patient 5	Infected wounds	1	36	50	*Enterobacter cloacae* complex, *Klebsiella oxytoca*, *Staphylococcus aureus*, *Raoultella ornithinolytica*	No	Unacid/Metronidazo Tazobactam
Patient 6	Subtrochanteric femur fracture	1	200	500	–	No	–
Patient 7	Open tibial spiral fracture 42A1c 1	1	90	100	–	No	Unacid
Patient 8	Traumatic opening of the prepatellar bursa	1	14	10	Negative	No	Unacid
Patient 9	Tibia fracture 42 A1.3	1	99	100	–	No	–
Patient 10	Open tibia fracture 42.A2.3	1	112	100	–	No	Tazobactam
Patient 11	Knee distortion	1	38	10	–	No	–
Patient 12	Partial rupture of the Lateral collateral ligament	1	35	10	–	No	–
Patient 13	Lateral tibial plateau fracture C1	1	90	150	–	No	–
Patient 14	Bilateral sacral fracture	2	170	150	–	Postoperative hematoma	Tazobactam
Patient 15	Abscess lower leg	2	46	50	*Citrobacter koseri*, *Klebsiella oxytoca*, *Enterococcus faecalis*, *Bacteroides fragilis*, *Staphylococcus epidermidis*	No	Tazobactam

There were no intra-hospital deaths among the patients. Nineteen patients were discharged home (or to family when their houses had been destroyed), whereas three patients were discharged to a rehabilitation clinic.

With limited resources in the coming weeks, many patients did not receive adequate medical care. Many pharmacies were destroyed, as were doctors' offices. As a result, patients did not receive medications or routine exams. Due to the destruction of their own homes and the loss of relatives, neighbours and friends, many patients were traumatised and unable to work. As a result of the limited medical care, these patients were only able to receive partial medical treatment and sick leave was not possible. This led to further problems for the victims with their employers, insurance companies and compensation.

As a result of the floods, several regional hospitals were partially or completely destroyed and patient care had to be suspended [6, 14]. Thanks to nationwide relief efforts and the support of health insurance and medical associations, medical care was partially restored.

## Case studies

### Case study I

**Fig. 2:**
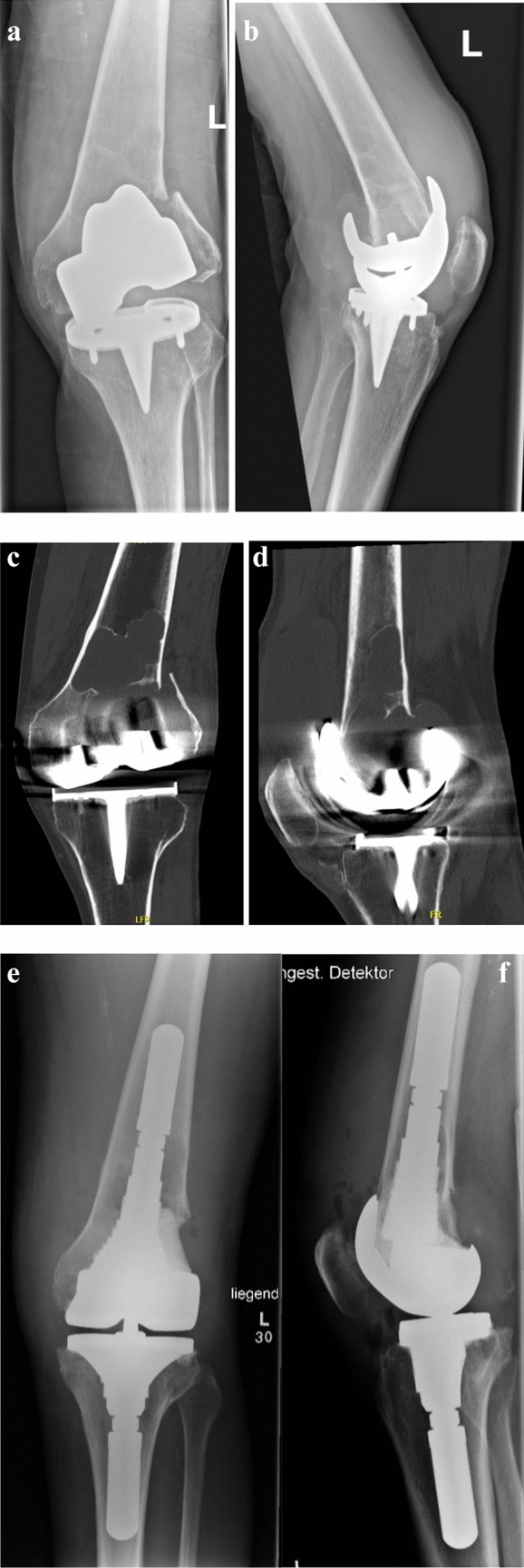
**a** Sixty-nine-year-old male patient with a dislocated periprosthetic distal femur fracture type Lewis–Rorabeck III. Technique: AP X-ray of the left knee joint. The femoral component seems loose and is dislocated. **b** Lateral X-ray of the knee joint. The distal femur shows multiple cystic lesions. **c**, **d** Digitally reconstructed computed tomography scan of the left knee joint in anterior and lateral views. The CT scan confirmed the loss of the femoral component and massive cystic formations in the proximal femur. Postoperative AP X-ray (**e**) and lateral view (**f**). The loosened knee replacement was removed and replaced with a cemented revision prosthesis (MUTARS^®^ GenuX^®^, Implantcast GmbH, Buxtehude, LS, Germany). The lateral fragment could not be refixed and was removed

A 69-year-old male patient tried to safeguard his house from the water and fell on a flooded staircase. The patient suffered a periprosthetic distal femur fracture typed as Lewis–Rorabeck III (Fig. 2). Because of the loosening of the total knee replacement (TKA) and massive osteolysis seen in an additional computed tomography (CT) scan, a revision-TKA was necessary. Full weight bearing and free range of motion were allowed after surgery. The patient was able to walk with crutches on the day after surgery, and he was dismissed 6 days after admission.

### Case study II

**Fig. 3:**
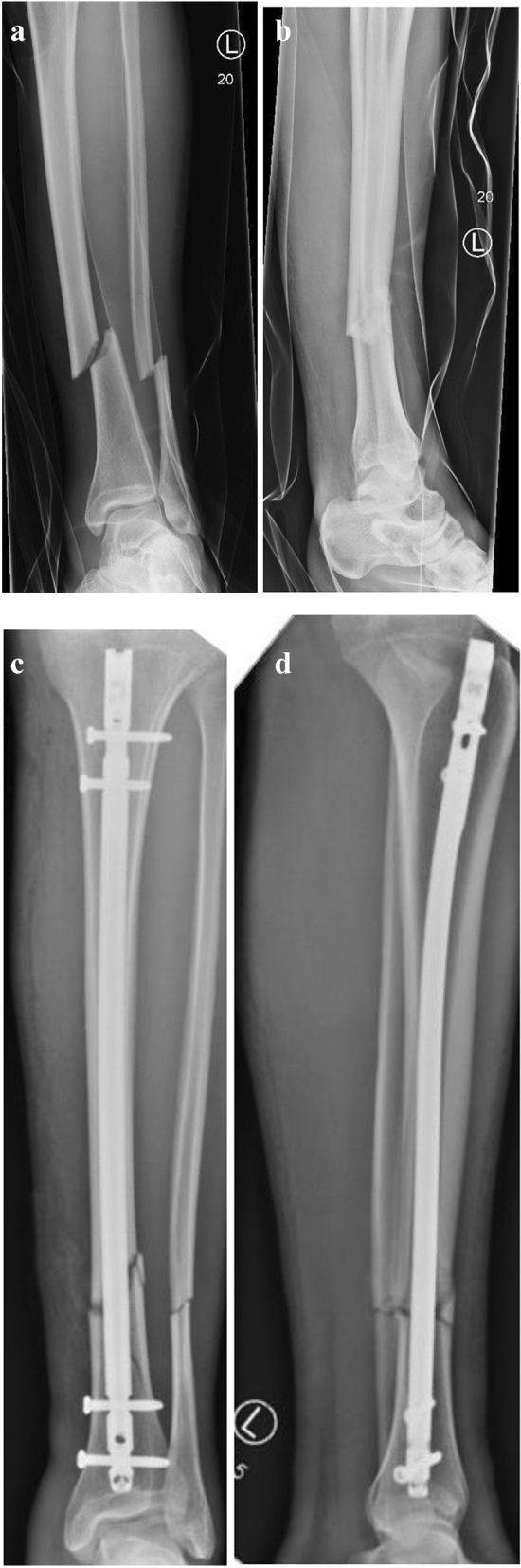
Thirty-two-year-old male patient with a closed fracture of the lower leg (AO 42B2) X-ray in ap (**a**) and lateral view (**b**). AP X-ray (**c**) and lateral (**d**) view after closed reduction and internal fixation via intramedullary nail (Tibia EXPERT™ nail, Synthes, West Chester, PA, USA)

A 32-year-old male volunteer of an aid organisation suffered a closed tibia fracture (AO 42B2) during clean-up works 6 days after the flood. His left leg was pinched by falling debris. After airborne transport of the patient to the hospital, a closed reposition and intramedullary nailing was performed on the same day the trauma occurred. (Fig. 3). Partial weight bearing and free range of motion were allowed after surgery. The patient was able to walk with crutches on the day after surgery and was transferred to a hospital close to his home.

## Discussion

This article gives an overview of the aftermath and the treatment of injured patients of the flash flood and mudslides occurring on 14 July 2021 in Germany. A total of 63 patients were treated in a level I trauma centre. Twenty operations were required, some of which were complex and covered the entire spectrum of orthopaedic and trauma surgery. Highly specialised centres are essential to mastering such events.

Luckily, the largest trauma centre was not affected in this case, and it was roughly 30 km away from the worst hit areas. Other hospitals, medical practices, and pharmacies were severely affected by the flood and had to cease treatment or operations, or alternatively, limit the available procedures to emergency interventions [8, 14]. Such closures can lead to higher concentrations in larger, more distant hospitals. For instance, Burger and Canton described numerous effects after Hurricane Katrina that led to a loss of power and air conditioning in an orthopaedic hospital [15]. The supply of fresh water stopped completely, resulting in the loss of all sanitation abilities. Furthermore, all communication channels and phone lines were down, limiting planning to transfer patients or organise supplies. Hospital personnel worked day and night to clean up the hospital and deal with the damage to their homes. The authors of that study stressed the need to save communication systems, such as satellite phones, as a key to managing a natural disaster. They also noted that most emergency plans and drills focus on bringing patients to hospitals in case of a disaster; however, plans to save or evacuate a clinic are at least as important. Although our institution was not directly affected, we noticed indirect effects, such as clinic personnel being unable to reach their workplace or needing to care for their homes and personal belongings. Furthermore, there was a higher patient volume because of compromised hospitals.

**Fig. 4:**
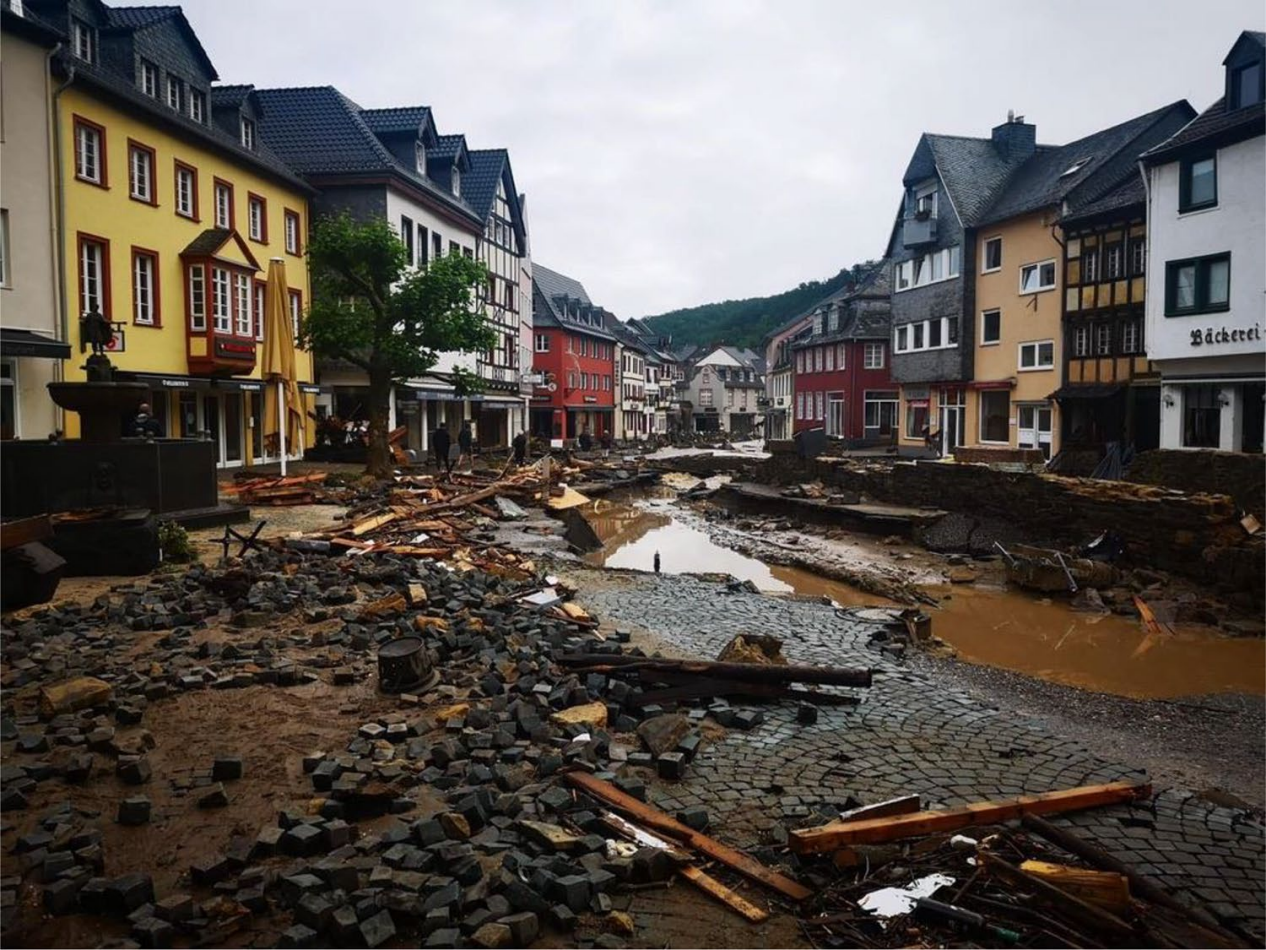
Representation of the destruction using the example of Bad Münstereifel on 16 July 2021

Another notable peculiarity was that besides directly affected patients, we saw a high number of injured patients even days and weeks after the event. Thirteen (59%) patients were treated due to injuries received directly during the flood. However, after the flash flood, villages, homes, and streets were full of mud, debris, and wreckage that needed to be removed (Fig. 4). As a result, many patients suffered severe wound infections because of contact with polluted water or were injured during clearing work (see case study II). None of the reported patients in this study suffered a polytrauma. The literature on comparable disasters and the proportion of polytrauma patients is very limited. Gao et al. analysed more than 2100 patients after an earthquake in China in 2008. Only 3% of them were polytrauma patients. In context of our data, survivors of natural disasters rarely appear to experience a polytrauma [16]. An important aspect here could be the limited on-site care. Since many of those who died were discovered only after the event, the chances of survival for polytrauma victims appear to be low.

The question of whether orthopaedic surgeons are prepared for such events or not, is not easily answered. On the one hand, there are established structures such as the Trauma Network and the MCI to be best prepared for disasters. In the end, it depends on the professional competence of the staff whether care can be provided. Therefore, staff are now sent annually to a Terror and Disaster Surgical Care (TDSC^®^) course at the Academy of Trauma Surgery (AUC) to deal with these variable situations. Additionally, regular training in Damage control surgery (DCS) is held for all staff. However, extensive staff training is needed at all hospitals to provide patient care during more severe disasters in the future.

The authors cannot sufficiently stress the importance of an interdisciplinary setting to master an unpredictable catastrophe such as the one described in this paper. In this event, patients showed numerous symptoms besides their injuries and needed treatment from different departments and disciplines. The patients showed signs of exhaustion and hypothermia, and they needed advisory support from multiple disciplines, such as internal medicine, neurosurgery, or oral and maxillofacial surgery.

Many people were exposed to polluted water for a long time. In close cooperation with the Department of Microbiology of our clinic, patients with wounds and contact with water received Tazobactam as an empiric antibiotic. Studies investigating previous floods in relation to soft tissue infections showed that most pathogens are bacterial, mainly comprising Gram-negative *Aeromonas* species or Gram-positive *Enterococcus* species [17, 18]. In our data collection, specimens were taken from five patients and pathogens were found in three. All three patients had mixed infection with samples positive for *Enterococcus* species.

Finally, we treated patients who had lost their homes, their jobs, and in some cases, friends and family members. Thus, all patients were offered psychological co-treatment.

Dealing with possible threats, such as extreme weather events, failure of infrastructures, terrorist attacks, or pandemics, requires networked action by all actors involved on both the government side and the side of private infrastructure operators [19]. Germany is a nation in which environmental disasters occur relatively rarely. Thus, most surgeons have never experienced a setting with limited resources or limited communication options. Nevertheless, a survey of physicians in the United States, a country facing far more natural disasters, found that only 61% felt prepared to handle a natural disaster or an outbreak of a major airborne infection. The rate of colleagues who felt prepared to handle a chemical, biological, radiological, nuclear, or explosive (CBRNE) incident was only 34% [20, 21]. Surgeons should be aware of and trained for an optimal disaster response when there is a substantial change in the level of care, which is something made necessary by a catastrophic disaster [22]. Although education and training are accepted to be integral to disaster preparedness, many currently taught practices are neither evidenced based nor standardised [23]. Core competencies need to be taught in aspects of disaster risk management, such as effective leadership, preparedness and risk reduction, emergency response, and post-disaster health system recovery [24, 25]. Such training programmes as disaster management and emergency preparedness (DMEP) or terror and disaster surgical care (TDSC) courses and regular drills may help improve patient care and safety [26, 27].

Our study has several limitations. Compared with global catastrophes, the flooding described was a minor event. Therefore, the number of patients included is also rather low. However, natural disasters have so far been rare in the Federal Republic of Germany, and few data are available overall. Furthermore, only a few studies by European colleagues have focused on trauma management after natural disasters; thus, the comparability of the discussed studies is limited.

## Conclusion

With rising numbers of casualties of natural disasters, it may be crucial for orthopaedic and trauma surgeons to gather more data about the management and pitfalls of the treatment of affected patients. Hospitals should review its emergency plans to determine whether they can be used in the event of natural catastrophes.
